# Dietary patterns and child, parental, and societal factors associated with being overweight and obesity in Vietnamese children living in Ho Chi Minh city

**DOI:** 10.1111/mcn.13514

**Published:** 2023-04-03

**Authors:** Thi My Thien Mai, Quoc Cuong Tran, Smita Nambiar, Danielle Gallegos, Jolieke C. Van der Pols

**Affiliations:** ^1^ School of Exercise and Nutrition Sciences Queensland University of Technology (QUT) Brisbane Queensland Australia; ^2^ Ho Chi Minh City Center for Disease Control Ho Chi Minh City Vietnam; ^3^ Department of Nutrition and Food Safety, Faculty of Public Health Pham Ngoc Thach Medical University Ho Chi Minh City Vietnam; ^4^ Woolworths Centre for Childhood Nutrition Research Queensland University of Technology (QUT) South Brisbane Queensland Australia

**Keywords:** childhood overweight and obesity, dietary pattern, life‐course nutrition, nutrition transition, obesity inequity, parental perception, urban

## Abstract

Childhood overweight and obesity are rapidly increasing in urban Vietnam. Dietary patterns are understudied for their association with obesity risk in these children, and it is unclear which parental and societal factors should be targeted in prevention efforts. The study assessed child characteristics, dietary patterns, parental and societal factors for associations with childhood overweight and obesity status in Ho Chi Minh City, Vietnam. A sample of 221 children aged 9–11 years was randomly selected from four Ho Chi Minh City primary schools. Weight, height and waist circumference were measured using standardized methods. Three 24‐h dietary recalls were collected from 124 children, which were used to assess dietary patterns using principal component analysis (PCA). Parents completed a questionnaire about child, parental and societal factors. The overall prevalence of obesity was 31.7% and of combined overweight and obesity 59.3%. Three main dietary patterns from 10 food groups were identified by PCA: traditional (grains, vegetables, meat and meat alternatives), discretionary (snacks and sweetened beverages), and industrialized (fast food and processed meat). Children with higher discretionary diet scores had higher odds of being overweight. Being a boy, screen time over 2 h/day, parental underestimation of child weight status, father's obesity, and household income in the lowest quintile were positively associated with childhood obesity. Future intervention programmes in Vietnam need to consider targeting children's unhealthy diets and parental perceptions of child weight status, as well as focusing on upstream approaches that reduce inequities contributing to childhood obesity and concomitant dietary patterns.

## Key messages

- The prevalence of overweight and obesity in children aged 9–11 years in Ho Chi Minh City remains high with nearly 60% of children either overweight or obese and one in three children obese.
- Children were consuming three main dietary patterns: traditional, discretionary and industrialized. All dietary patterns were associated with varying magnitudes of childhood weight status (overweight, obesity and elevated waist‐to‐height ratio) although this was mostly attenuated after multivariable adjustment.
- Parental underestimation of child weight status was strongly associated overweight and obesity.
- There is a shift in the association of childhood obesity with income in Vietnam. Previous evidence indicated that children living in families with the highest incomes had an increased risk of obesity. However, evidence from our study that provided more recent data indicates that children living in households with lower incomes now have become more likely to be obese compared to children from higher income families.

## 1 INTRODUCTION

Overweight and obesity among children has rapidly increased in Vietnam, particularly in primary school children in urban areas (Mai et al., 2020b; Ngan et al., 2018). According to the National Institute of Nutrition, the prevalence of overweight and obesity among urban school‐aged children has doubled from 9% to almost 20% over the last 10 years between 2010 and 2020 (National Institute of Nutrition, 2021). Children with obesity have a higher risk of developing cardiovascular diseases, diabetes, psychological and mental health disorders, and they have a higher risk of being obese in adulthood (Reilly & Kelly, 2010). Childhood overweight and obesity is a major global public health issue and is attributable to biological, economic, cultural and societal factors at the household, local and national levels. In recent years, it has become increasingly clear that commercial determinants of health through food marketing, industry lobbying, corporate social responsibility strategies and food supply chain issues play a major role in the rapid increases in childhood obesity seen in many Asian countries (Kickbusch et al., 2016). Such factors influence the resources and practices related to nutrition and activities for children (Harrison et al., 2011).

There is some evidence that in Vietnam, risk factors for childhood obesity occur at several levels including the child level (being a boy, going to school by motorbike/car/bus, eating confectionary, spending less time for sports, less frequency of having breakfast at home); familial level (parental obesity, living with only father) and at the societal level (higher parent's education level, mother's employment) (Le & Dinh, 2022; Ngan et al., 2018; Pham et al., 2020).

In addition, Vietnam is moving through a nutritional transition due to rapid changes in economic development, urbanization and the increased consumption of unhealthy diets which may contribute to increases in childhood obesity (Harris et al., 2020; Harris et al., 2022). Rapid changes to food availability and income can profoundly impact the factors that contribute to childhood obesity. Given this rapidly changing context in Vietnam, more evidence regarding risk factors for overweight and obesity in primary school‐aged children is needed so that comprehensive programmes to tackle the high prevalence of overweight and obesity in urban Vietnam can be developed.

Few Asian countries have explored dietary patterns in school‐aged children and their association with overweight and obesity, with most studies in the region originating from China (Choy et al., 2021). Among Chinese school‐aged children, specific dietary patterns such as traditional North or modern pattern were associated with risk of obesity (Zhang et al., 2015; Zhen, 2018).

Parental perception of child weight status has been identified as a risk factor for childhood obesity. Globally, about 55% of caregivers of children underestimated their child's weight status (Alshahrani et al., 2021), with estimates from Asian parents ranging from 30% to 90% (Park, 2017). Children whose parents underestimated their weight had a higher odd of being obese (Alshahrani et al., 2021; McKee et al., 2016). This underestimation, in part, is due to the effects of the nutrition transition and changes to food access that has impacted many countries in Asia. After long periods of national hunger, an overweight child is a sign of accessing adequate food and is potentially a proxy for wealth and health (Do et al., 2016). However, the exploration of children's dietary patterns, and parental factors such as child weight status perceptions and parental self‐efficacy to influence a child's dietary and physical activity practices, have not often been studied in primary school‐aged children in Vietnam. Therefore, this study aimed to investigate children's dietary patterns and child, parental, societal factors associated with overweight and obesity in a sample of children in Ho Chi Minh City, Vietnam.

## 2 METHODS

### 2.1 Study design and study population

This study was part of a cross‐sectional study to examine the reproducibility and validity of short dietary questions in children 9–11 years old in Ho Chi Minh City, Vietnam (Mai et al., 2022). A sample size of 216 participants was required to perform univariate logistic regression to explore relationships between weight status, waist‐to‐height ratio, and selected determinants (prevalence of obesity at 30%; 95% power and odds ratio [OR] of 2.0) (Hsieh, 1989; Mai et al., 2020b). Four primary schools were randomly selected from four areas in Ho Chi Minh City, including urban‐wealthier districts, urban‐less‐wealthier districts, emerging urban districts, and rural districts located within the city boundaries. In each school, all students in fifth grade were sent a participant information sheet and invited to participate in the study. Children with completed consent and assent forms, who met the inclusion criteria (children were aged 9–11 years old, both parent and child agreed to participate in the study) were included in the study.

### 2.2 Variables

All children had their weight, height and waist circumference measured twice at school by a trained health officer using the standard protocol (Word Health Organization, 2008) and presented elsewhere (Mai et al., 2022). Overweight and obesity were determined using WHO's criteria for children from 5 to 19 years old: overweight was defined by BMI‐for‐age *Z*‐score > +1SD (WHO)(de Onis, 2007), obesity was defined by BMI‐for‐age *Z*‐score > +2SD (WHO) (de Onis, 2007). In addition, waist‐to‐height ratio was calculated with a cut‐off of 0.5 identifying cardiovascular risk (Mai et al., 2020a).

To examine the contributing factors to childhood obesity, parents completed a self‐administered questionnaire that was sent home with children. The questionnaire used closed‐ended questions structured into two sections: information about the family and child and parental perceptions of their child's body weight status. Information collected about the child included age, sex, birthweight, puberty status, level of physical activity, screen‐time and receiving pocket money. In relation to physical activity, two questions previously validated were asked about the number of days in the last week and in a usual week that the child had at least 60 min of activity that increased heart rate and caused puffing (Hardie Murphy et al., 2015). To improve the accuracy in estimating screen time, two close‐ended questions were asked about the number of hours per day that the child watched a screen (television/mobile phone/computer/tablet) during weekdays and during the weekend (Singh et al., 2011).

Family‐level information collected related to family structure, birth order of the child, number of adults in the household, number of children in the household, parent (father and mother) education level, parent employment status, parent self‐reported weight and height, household income. The ratio of children per adult in the household was calculated by dividing the number of children by the number of adults in the household (which could include grandparents if they lived in the same household). To identify parental weight status, self‐reported weight and height was used to calculate BMI. The specific cut‐off of 25 kg/m^2^ for Asian populations was used to identify adults with obesity (WHO & IOTF/IASO, 2000). Household income was categorized into quintiles according to the monthly income of Ho Chi Minh citizens in 2019 (Ho Chi Minh City Statistical Office, 2019).

The Health Belief Model (Baranowski et al., 2003) was used to underpin the examination of parental perception of their child's nutritional status, about the seriousness of childhood overweight and obesity, and about their self‐efficacy in influencing child's dietary practices and physical activities. The motivation for changing the child's behaviours was stimulated by the perceived risk and seriousness of childhood obesity and the confidence of parents to control the child's behaviours (Daddario, 2007). Parental perception of their child's weight status was measured by one text‐based question (Tompkins et al., 2015) and one image‐based question (Truby & Paxton, 2002; Truby & Paxton, 2008). Parental self‐efficacy was evaluated by using a 14‐item questionnaire about parental self‐efficacy to influence a child's dietary practices (8 items) and physical activities (6 items) (Norman et al., 2018). The parental self‐efficacy tool had not previously been validated in an Asian population. However, in the pilot study, the internal consistency reliability of this questionnaire was high with a Cronbach's *α* of 0.95. The intra‐class correlation from the first to the second administration 1 week apart was 0.90 (0.77–0.95).

To examine children's intake from different food groups, frequency of intakes from food groups were derived from three nonconsecutive 24‐h recalls. To adjust for frequency intakes during weekdays and weekends, the intake frequencies from three 24‐h recalls were calculated using the following equation: average frequency of weekday multiplied by 5 plus frequency of weekend multiplied by 2, the total number was divided by 7. All 24‐h recalls were reported by the child to a trained interviewer at school using food pictures (including popular street foods available in Ho Chi Minh city) and common utensils to assist with portion sizes. Rulers were used to assist with estimation of food dimensions. Children who under‐ and over‐reported their dietary intakes were identified using Goldberg's method (Elliott et al., 2011; Goldberg et al., 1991), using a predicted basal metabolic rate in the context of overweight and obesity existing in the study population (Maffeis et al., 1993; Molnár et al., 1995). In total, 19 out of 163 participants who under‐ or over‐reported energy intake from three 24‐h recalls were excluded from the analysis, resulting in a total of 144 participants with available dietary data. The characteristics of under and over‐reporters are presented in Supporting Information: Table S1.

### 2.3 Analysis

Descriptive data are presented as the number of participants and percentages for categorical variables and mean and standard deviation for continuous variables. Student's *T* tests evaluated differences between means for continuous variables, while *χ* ^2^ tests was used to evaluate differences in percentages among categorical variables.

Principle component analysis (PCA) was applied to derive dietary patterns in children from frequency intakes of food groups, using data from the three 24‐hour recalls collected over a week. To improve interpretability, five core‐food groups (cereals, vegetables, fruits and fruit juices, meat and alternatives, dairy and milk products) and five noncore food groups (sweetened beverages, snacks and discretionary foods, fast foods, instant noodles, processed meat) were created. Intake of these food groups was summarised as the frequency of intake per day. Details of the food group characteristics are presented in Supporting Information: Table S2. Using PCA, 10 dietary patterns were derived from the 10 food groups. Three major dietary patterns were retained, using a scree plot level‐off and eigenvalue >  1. Varimax rotation was then applied to retained components to obtain an orthogonal solution. Scores for each component were created by summing the observed intakes of component food items weighted by the factor loading. The patterns were labelled traditional, discretionary and industrialized based on the food groups that had the highest factor loadings in each pattern (Supporting Information: Table S3). Scores for each pattern were categorised into three equal groups using tertile cut‐offs for further analysis.

The potential risk factors for overweight, obesity, overweight and obesity (combined), and elevated waist‐to‐height ratio (>0.5) were examined using logistic regression with healthy weight and underweight (combined) as the reference group. All variables including children's characteristics, sociodemographics characteristics, and parental perceptions were individually tested for association with overweight, obesity, combined overweight and obesity and elevated waist‐to‐height ratio. Multivariate imputation was then applied for variables with missing values in the adjusted mutivariate logistic regression model. In addition, the analysis was repeated with the sub‐sample of children with dietary data to examine the association between dietary patterns and child nutritional status. Dietary patterns were associated with overweight, obesity, and elevated waist‐to‐height ratio, thus associated factors for overweight, obesity, and elevated waist‐to‐height ratio were presented for this sub‐sample in a separate table of results. All models without dietary data were adjusted for physical activity level. In addition, all models with dietary patterns were adjusted for mean energy intake, which was calculated from the three 24 h recalls, and for physical activity level. Sensitivity analyses were performed by comparing models with and without imputation. The results were presented as OR and 95% confidence interval (CI) from the imputation model as there were no differences between the models with and without imputation. Statistical significance was defined as *p* < 0.05. Analyses were conducted using STATA 17 (StataCorp LLC).

The study was conducted in accordance with the Declaration of Helsinki and approved by the Queensland University of Technology Human Research Ethics Committee.

## 3 RESULTS

A total of 250 out of 712 (35.1%) children who were invited to participate in this study returned two consent forms. Of the 250 questionnaires sent, 226 were returned to the research assistant. After checking for missing data, five questionnaires with more than 80% missing responses were excluded, resulting in available data on 221 participants for the analysis. Dietary data were available for 124 participants (56.1%).

### 3.1 Participants' characteristics

The prevalence of combined overweight and obesity was 59.3% with the prevalence of obesity being 31.7% and the prevalence of elevated waist‐to‐height ratio being 33.9% (*N* = 221). In a subgroup of 124 participants with dietary data, these figures were 57.6%, 27.4% and 27.4%, respectively. Only seven children (3.2%) were underweight and these children were grouped with healthy‐weight children for all subsequent analyses. The mean age was 10.6 ± 0.5 years and 45.7% of participants were boys. Ninety‐two percent of participants had a birthweight between 2500 and 4000 g. About 23% of children were reported to have reached puberty and most of these were girls (82%). In addition, 74% of participants did not meet the physical activity guideline and 58% of participants exceeded the screen‐time recommendation. Characteristics of participants are presented in Table 1.

**Table 1:** General characteristics of participants and associations with body weight status (*N* = 221).

Characteristics of participants	Total participants	Healthy weight and underweight	Overweight	Obese	Overweight and obesity	Elevated WHtR
Total (*n* = 221)		90 (40.7)	61 (27.6)	70 (31.7)	131 (59.3)	75 (33.9)
Subsample dietary intake (n = 124)		53 (42.7)	37 (29.9)	34 (27.4)	71 (57.3)	34 (27.4)
Area (*n* = 221)						
Rural district	53 (24.0)	21 (39.6)	11 (20.8)	21 (39.6)	32 (60.4)	21 (39.6)
Urban district	168 (76.0)	69 (41.1)	50 (29.8)	49 (29.2)	99 (58.9)	54 (32.1)
Age (*n* = 221)	10.6 (0.5)	10.6 (0.5)	10.6 (0.6)	10.6 (0.5)	10.6 (0.5)	10.6 (0.5)
Sex (*n* = 221)						
Boys	101 (45.7)	32 (31.7)	20 (19.8)	49 (48.5)	69 (68.3)	48 (47.5)
Girls	120 (54.3)	58 (48.3)	41 (34.2)	21 (17.50)**	62 (51.7)**	27 (22.5)**
Ethnicity (*n* = 220)						
Kinch	211 (95.9)	85 (40.3)	58 (27.5)	68 (32.2)	126 (59.7)	73 (34.6)
Others (Chinese, Khmer)	9 (4.1)	5 (55.6)	2 (22.2)	2 (22.2)	4 (44.4)	2 (22.2)
Birthweight (*n* = 192)						
<2500 g	8 (4.2)	4 (50)	1 (12.5)	3 (37.5)	4 (50)	3 (37.5)
2500–<4000 g	176 (91.7)	75 (42.6)	48 (27.3)	53 (30.1)	101 (57.4)	58 (33.0)
≥4000 g	8 (4.2)	3 (37.5)	0 (0.0)	5 (62.5)	5 (62.5)	5 (62.5)
Puberty (*n* = 213)						
No	163 (76.5)	69 (42.3)	42 (25.8)	52 (31.9)	94 (57.7)	58 (35.6)
Yes	50 (23.5)	20 (40)	16 (32)	14 (28)	30 (60)	13 (26.0)
Sufficient Physical Activity (*n* = 211)						
No	156 (73.9)	62 (39.7)	46 (29.5)	48 (30.8)	94 (60.3)	54 (34.6)
Yes	28 (13.3)	14 (50)	3 (10.7)	11 (39.3)	14 (50)	10 (35.7)
Unknown	27 (12.8)	10 (37)	8 (29.6)	9 (33.3)	17 (63)	9 (33.3)
Screen time (*n* = 215)						
<2 h/day	91 (42.3)	42 (46.2)	27 (29.7)	22 (24.2)	49 (53.9)	23 (25.3)
≥2 h/day	124 (57.7)	47 (37.9)	31 (25)	46 (37.1)	77 (62.1)	51 (41.1)*
Pocket money (*n* = 215)						
No	74 (34.4)	31 (41.9)	16 (21.6)	27 (36.5)	43 (58.1)	24 (32.4)
Yes	141 (65.6)	57 (40.4)	41 (29.1)	43 (30.5)	84 (59.6)	51 (36.2)

*Note*: *χ* ^2^ test examined the difference between children with overweight, with obesity, or with overweight and obesity and children with healthy weight and underweight; WHtR waist to height ratio. Data presented as *n*(%) except for age was presented as mean(SD).

* *p* < 0.05

** *p* < 0.001.

Comparisons between children with overweight, obesity, combined overweight and obese, and children with healthy weight and underweight are also presented in Table 1. Obesity, combined overweight and obesity, or elevated waist‐to‐height ratio was significantly higher in boys than in girls (48.5% vs. 17.5%, *p* < 0.001, 68.3% vs. 51.7%, *p* < 0.05, 47.5% vs. 22.5%, *p* < 0.001, respectively). Overweight was higher in girls than boys but this difference was not stastistically (34.2% vs. 19.8%, *p* > 0.05). In addition, children who were spending at least 2 h/day watching screens had higher rates of elevated waist‐to‐height ratios than those spending less than 2 h of watching screens (41.1% vs. 25.3%, *p* < 0.05).

### 3.2 Sociodemographic characteristics

Data on sociodemographic characteristics are presented in Table 2. Nearly 60% of the children lived in a parent(s)‐with‐children family structure and the majority of children (73.4%) were either the youngest or the oldest in the family. Nearly one‐half of fathers (45.1%) and about one‐third of mothers (29.6%) had a college/university or postgraduate education. Fathers were more likely than mothers to be in skilled employment (70% vs. 53.5%, *p* > 0.05). About one‐quarter (26%) and two‐thirds (66%) of fathers and mothers, respectively, were overweight. Household income groups were fairly represented in this sample (Table 2).

**Table 2:** Socio‐demographics characteristics (*N* = 221).

Socio‐demographics characteristics	Total participants	Healthy weight and underweight	Overweight	Obese	Overweight and obesity	Elevated WHtR
Family structure (*n* = 217)						
Nuclear family (parents and child)	128 (59)	50 (39.1)	37 (28.9)	41 (32.0)	78 (60.9)	47 (36.7)
Others (grandparents, parents, child)	89 (41)	40 (44.9)	21 (23.6)	28 (31.5)	49 (55.1)	28 (31.5)
Birth order (*n* = 218)						
Oldest and youngest	160 (73.4)	65 (40.6)	41 (25.6)	54 (33.8)	95 (59.4)	59 (36.9)
Middle	58 (26.6)	25 (43.1)	18 (31)	15 (25.9)	33 (56.9)	15 (25.9)
Ratio of child per adults (*n* = 213)						
One child per two adults or less	71 (33)	27 (39.1)	13 (18.8)	29 (42)	42 (60.9)	28 (40.6)
More than one child per two adults	144 (67)	62 (43.1)	44 (30.6)	38 (26.4)	82 (56.9)	46 (32.0)
Father's education (*n* = 202)						
Primary school or lower	21 (10.4)	8 (38.1)	4 (19.1)	9 (42.9)	13 (61.9)	11 (52.4)
Secondary school	37 (18.3)	15 (40.5)	12 (32.4)	10 (27)	22 (59.5)	13 (35.1)
High school and vocational training	53 (26.2)	19 (34.6)	10 (18.9)	20 (37.7)	30 (56.6)	20 (37.7)
College/university/postgraduates	91 (45.1)	25 (41.7)	29 (31.9)	26 (28.6)	55 (60.4)	27 (29.7)
Mother's education (*n* = 203)						
Primary school or lower	60 (29.6)	24 (40)	14 (23.3)	22 (36.7)	36 (60)	21 (35.0)
Secondary school	28 (13.8)	15 (53.6)	7 (25.0)	6 (21.4)	13 (46.4)	8 (28.6)
High school and vocational training	55 (27.1)	18 (32.7)	13 (23.6)	23 (41.8)	36 (65.5)	22 (40.0)
College/university/postgraduates	60 (29.6)	24 (40)	19 (31.7)	16 (26.7)	35 (58.3)	22 (36.7)
Father's employment (*n* = 176)						
Unskilled employment	53 (30.1)	16 (30.2)	18 (34)	19 (35.9)	37 (69.8)	23 (43.4)
Skilled employment	123 (69.9)	55 (44.7)	34 (27.6)	34 (27.6)	68 (55.3)	36 (29.3)
Mother's employment (*n* = 202)						
Unskilled employment	94 (46.5)	37 (39.4)	26 (27.7)	31 (33)	57 (60.6)	31 (33.0)
Skilled employment	108 (53.5)	45 (41.7)	30 (27.8)	33 (30.6)	63 (58.3)	37 (34.3)
Father's BMI (BMI ≥ 25) (*n* = 198)						
No	147 (74.2)	68 (46.3)	40 (27.2)	39 (26.5)	79 (53.7)	48 (32.6)
Yes	51 (25.8)	13 (25.5)	15 (29.4)	23 (45.1)*	38 (74.5)*	19 (37.3)
Mother's BMI (BMI ≥ 25) (*n* = 197)						
No	66 (33.5)	29 (43.9)	23 (34.9)	14 (21.2)	37 (56.1)	19 (28.8)
Yes	131 (66.5)	51 (38.9)	32 (24.4)	48 (36.6)	80 (61.1)	48 (36.6)
Household's income (VND) (*n* = 208)						
<5 million (1st quintile)	30 (14.4)	11 (36.7)	5 (16.7)	14 (46.7)	19 (63.3)	14 (46.7)
5–<10 million (2nd quintile)	43 (20.7)	18 (41.9)	15 (34.9)	10 (23.3)	25 (58.1)	11 (25.6)
10–<15 million (3rd quintile)	50 (24.0)	19 (38)	11 (22.0)	20 (40.0)	31 (62.0)	20 (40.0)
15–<20 million (4th quintile)	28 (13.5)	10 (35.7)	7 (25.0)	11 (39.3)	18 (64.3)	10 (35.7)
20 million or above (5th quintile)	57 (27.4)	27 (47.4)	19 (33.3)	11 (19.3)*	30 (52.6)	16 (28.1)

*Note*: *χ* ^2^ test the difference between children with overweight, with obesity, or with overweight and obesity and children with healthy weight and underweight. Data were presented as *n*(%).

* *p* < 0.05.

Children whose father was obese had a higher prevalence of obesity and combined overweight and obesity than those with nonobese father (45.1% vs. 26.5%, *p* < 0.05 and 74.5% vs. 53.7%, *p* < 0.05, respectively). This trend was similar to mothers' BMI but associations were not statistically significant. Households in the fifth quintile income had significantly lower proportions of children with obesity compared with those living in households in the first income quintile (19.3% vs. 46.7%, *p* < 0.05).

### 3.3 Parental perceptions

Table 3 presents parental perception of their children's weight status and their perceived self‐efficacy to influence their child's dietary practices and physical activity. Around half of the parents who completed the questionnaire (of which 73% were mothers and 22% fathers) perceived that their child was about the right weight using the text‐based question, or in an acceptable weight range when using the image‐based question. However, most parents underestimated their child's weight status, both in the text‐based question (56.9%) and the image‐based question (61.1%). In addition, 82.5% (text‐based question) and 74.8% (image‐based question) of children whose parents underestimated their child's weight status were overweight or obese. The proportion of parents who correctly estimated their children's weight status was equal between the text‐based and image‐based question (39.8% and 38.0%, respectively). However, the proportion of parents who correctly categorised their child as obese was higher when the image‐based question was used than when they used the text‐based question (24% vs. 12%).

**Table 3:** Parental perception of their child's nutritional status, seriousness of childhood overweight and obesity and their self‐efficacy to influence children's eating behaviours and physical activity (*N* = 221).

Parental perception and self‐efficacy	Total participants	Healthy weight and underweight	Overweight	Obese	Overweight and obesity	Elevated WHtR
*Parental perception on child's status using description by wording* (*n* = 211)**						
Very underweight	3 (1.4)	3 (100)	0 (0.0)	0 (0.0)	0 (0.0)	0 (0.0)
A little underweight	25 (11.9)	23 (92)	1 (4.0)	1 (4.0)	2 (8.0)	1 (4.0)
About the right weight	114 (54.0)	56 (49.1)	41 (36.0)	17 (14.9)	58 (50.9)	24 (21.1)
A little overweight	57 (27.0)	3 (5.3)	15 (26.3)	39 (68.4)	54 (94.7)	39 (68.4)
Very overweight	12 (5.7)	2 (16.7)	0 (0.0)	10 (83.3)	10 (83.3)	8 (66.7)
*Difference between perception on child's nutritional status by wording and actual child's nutritional status* (*n* = 211)**						
Under‐ estimated	120 (56.9)	21 (17.5)	42 (35.0)	57 (47.5)	99 (82.5)	56 (46.7)
Matched	84 (39.8)	59 (70.2)	15 (17.9)	10 (11.9)	25 (29.8)	15 (17.9)
Overestimated	7 (3.3)	7 (100.0)	0 (0.0)	0 (0.0)	0 (0.0)	1 (14.3)
*Parental perception on child's status using body image scale (n = 208)* **						
Thinness	42 (20.2)	38 (90.5)	4 (9.5)	0 (0.0)	4 (9.5)	0 (0.0)
Acceptable	106 (51.0)	47 (44.3)	43 (40.6)	16 (15.1)	59 (55.7)	23 (21.7)
Overweight	41 (19.7)	1 (2.4)	8 (19.5)	32 (78.1)	40 (97.6)	33 (80.5)
Obese	19 (9.1)	0 (0.0)	0 (0.0)	19 (100)	19 (100.0)	15 (78.9)
*Difference between perception on child's weight status via body image scale vs actual child's nutritional status (n = 208)* **						
Underestimated	127 (61.1)	32 (25.2)	47 (37.0)	48 (37.8)	95 (74.8)	50 (39.4)
Matched	79 (38.0)	52 (65.8)	8 (10.1)	19 (24.1)	27 (34.2)	21 (26.6)
Overestimated	2 (1.0)	2 (100.0)	0 (0.0)	0 (0.0)	0 (0.0)	0 (0.0)
*Parentals perceived the seriousness of childhood overweight and obesity (n = 205)*						
Not serious	25 (12.2)	10 (40.0)	11 (44.0)	4 (16.0)	15 (60.0)	9 (36.0)
Somewhat serious	107 (52.2)	45 (42.1)	25 (23.4)	37 (34.6)	62 (57.9)	37 (34.6)
Very serious	73 (35.6)	28 (38.4)	21 (28.8)	24 (32.9)	45 (61.6)	24 (32.9)
*Parental's self‐efficacy* (*n* = 161)†						
Total score (max = 140)	93 (30.1)	95 (29.4)	102 (24.6)	85 (33.3)	93 (30.7)	89 (31.1)
Eating score (max = 80)	56 (18.1)	57 (18.0)	61 (16.5)	52 (18.7)	56 (18.3)	53 (18.4)
PA score (max = 60)	37 (14.9)	38 (14.1)	41 (11.1)	34 (17.8)	37 (15.5)	34 (16.0)

*Note*: *χ* ^2^ test of the difference between children with obesity, with overweight, with overweight and obesity and children with healthy weight and underweight.

** *p* < 0.001 for all group comparisons relating to this variable.

^†^ This variable was presented as mean score and SD.

Around one‐third (36.6%) of parents perceived that overweight and obesity were very serious conditions. Half of the parents (52.2%) indicated these conditions were somewhat serious. However, among those who believed that childhood overweight and obesity was not serious, 40% had a child in the healthy or underweight range, 44% had a child who was overweight and 16% had a child who was obese.

The mean score for parental self‐efficacy was 93.0 ± 30.1 out of 140. Although there were no statistically significant differences, parents of children who were obese or underweight had lower self‐efficacy scores (85 and 78, respectively) compared with parents of children with healthy weight (96) or overweight (102). This trend was similar for the subscore of self‐efficacy regarding parent's ability to influence their child's dietary or physical activity practices.

### 3.4 Dietary patterns

Three major dietary patterns were identified in this sample (Supporting Information: Table S3). The first major dietary pattern labelled ‘traditional’ included high factor loadings for grains, vegetables, meat and alternatives, and low factor loading for instant noodles. The next dietary pattern was labelled as ‘discretionary’ and was characterised by consumption of snacks such as salty snacks, sweets and sweetened beverages (including milk with added sugar). The final major dietary pattern was ‘industrialized’ which had high factor loadings for fast food, processed meats and low factor loadings for dairy products such as plain milk, plain yogurt and cheese.

### 3.5 Factors associated with childhood overweight and obesity

Risk factor associations were examined separately for overweight, obesity, overweight and obesity (combined), and elevated waist‐to‐height ratio (Table 4). For associations with overweight and obesity, being a girl (OR (95% CI) = 0.50 (0.29–0.86), *p* < 0.05), and having a parent able to correctly estimate a child's weight status (OR (95% CI) = 0.17(0.09–0.31) were negatively associated with overweight and obesity. Children who had a father with obesity were significantly more likely to be overweight and obese compared to those with a father without obesity (OR (95% CI) = 2.52(1.24–5.11), *p* < 0.05). After adjustment for confounding variables, father's BMI and parental perception of child weight status were independently associated with overweight and obesity (OR (95% CI) = 2.57(1.13–5.85), *p* < 0.05 and OR (95% CI) = 0.16(0.09–0.30), *p* < 0.001). For children with overweight, the correct parental perception of child weight status was a protective factor (OR (95% CI) = 0.10(0.04–0.25), *p* < 0.001). For children with obesity, sex (being a girl) (OR (95% CI) = 0.26(0.12–0.56), *p* < 0.001), father's obesity (OR (95% CI) = 3.72(1.37–10.07), *p* < 0.05), correct parental perception of child weight status (OR (95% CI) = 0.20(0.09–0.45), *p* < 0.001) and household income ≥20 million VND/month (OR (95% CI) = 0.23(0.07–0.82), *p* < 0.05) were individually associated with obesity and remained so after adjustment. For children with elevated waist‐to‐height ratio, sex (being a girl) (OR (95% CI) = 033(0.18–0.60), *p* < 0.001), at least 2 h per day of screen watching (OR (95% CI) = 2.40(1.25–4.60), *p* < 0.05), correct parental perception of child weight status (OR (95% CI) = 0.45(0.23–0.88), *p* < 0.05) were also individually associated with elevated waist‐to‐height ratio and this remained after adjustment.

**Table 4:** Contributing factors to childhood overweight and obesity in school children 9–11 years old in urban Vietnam (*N* = 221).

Contributing factors	Overweight and obesity (*n* = 131)		Overweight (*n* = 61)	Obesity (*n* = 70)		Elevated WHtR (*n* = 75)	
	OR (95%CI)	aOR(95%)	OR (95%CI)	OR (95%CI)	aOR(95%)	OR (95%CI)	aOR(95%)
*Child's characteristics*							
Sex (girls vs boys)	0.50 (0.29–0.86)*	0.53 (0.29–1.01)	1.13 (0.57–2.25)	0.24 (0.12–0.46)**	0.26 (0.12–0.56)**	0.32 (0.18– 0.57)**	033 (0.18–0.60)**
Screen time (≥2 h vs < 2 h)	1.4 (0.8–2.4)		1.02 (053–2.00)	1.87 (0.97–3.60)		2.06 (1.14– 3.74)*	2.40 (1.25–4.60)*
*Parent's characteristics*							
Father's BMI (BMI ≥ 25 vs BMI < 25)	2.52 (1.24–5.11)*	2.57 (1.13–5.85)*	1.96 (0.85–4.54)	3.08 (1.41–6.77)*	3.72 (1.37–10.07*	1.22 (0.63–2.40)	
Parental perception on child's nutritional status (matched & overestimated vs underestimated)	0.17 (0.09–0.31)**	0.16 (0.08–0.30)**	0.10 (0.04–0.25)**	0.24 (0.12–0.47)**	0.20 (0.09–0.45)**	0.54 (0.29–0.99)*	0.45 (0.23–0.88)*
*Family's characteristics*							
*Household's income quartiles (VND)*							
5–<10 vs. <5 million	0.80 (0.31–2.10)		1.83 (0.52–6.46)	0.44 (0.14–1.32)	0.37 (0.11–1.31)	0.39 (0.15–1.06)	
10–< 15 vs. <5 million	0.94 (0.37–2.41)		1.27 (0.35–4.64)	0.83 (0.30–2.27)	0.94 (0.29–2.98)	0.76 (0.31–1.90)	
15–<20 million vs. <5 million	1.04 (0.36–3.04)		1.54 (0.37–6.45)	0.86 (0.27–2.77)	0.72 (0.19–2.79)	0.63 (0.22–1.82)	
20 million or above vs. <5 million	0.64 (0.26–1.59)		1.55 (0.46–5.19)	0.32 (0.11–0.92)*	0.23 (0.07–0.82)*	0.44 (0.18–1.79)	

*Note*: OR, odd ratio (reference group is healthy/underweight children), aOR, adjusted odd ratio (adjusted for physical activity and for each others).

* *p* < 0.05

** *p* < 0.001.

Additional analysis of risk factors associated with overweight, obesity, combined oveweight and obesity, and elevated waist‐to‐height ratio in a subsample of children with dietary data (*N* = 124) are presented in Table 5. In univariate analyses, dietary patterns were associated with variable magnitudes of childhood weight status. High traditional pattern scores were associated with obesity (OR (95% CI) = 3.6(1.22–10.64)), whereas high discretionary pattern scores were associated with overweight (OR (95%CI) = 3.11(1.04–9.3)), and high industrialized pattern scores were associated with elevated waist‐to‐height ratio (OR (95% CI) = 2.88(1.02–8.09)). After adjustment for energy intake, physical activity and other confounding factors, only the discretionary pattern remained associated with being oveweight (2nd vs. 1st tertile, OR (95%CI) = 4.76(1.17–19.26)). In addition, after multivariable adjustment (Table 5), the correct parental perception of child weight status remained associated with overweight OR (95%CI) = 0.06(0.02–0.21) and obesity (OR (95% CI) = 0.24(0.08–0.74), and sex (being a girl) remained associated with obesity OR (95%CI) = 0.19(0.06–0.57) and elevated waist‐to‐height ratio (OR (95% CI) = 0.23(0.09–0.59).

**Table 5:** Contributing factors to childhood overweight and obesity in school children 9–11 years old in urban Vietnam in a sub sample with dietary data (*N* = 124).

Contributing factors	Overweight (*n* = 37)		Obesity (*n* = 34)		Elevated WHtR (*n* = 34)	
	OR (95%CI)	aOR(95%)	OR (95%CI)	aOR(95%)	OR (95%CI)	aOR(95%))
*Child's characteristics*						
Sex (girls vs. boys)	1.37 (0.56–3.30)		0.24 (0.92–0.60)*	0.19 (0.06‐0.57)*	0.21 (0.09–0.50)**	0.23 (0.09–0.59)*
Diet (traditional pattern)						
2nd tertile vs. 1st tertile	1.43 (0.53–3.84)		1.38 (0.44–4.29)	0.96 (0.26‐3.55)	1.18 (0.42–3.29)	
3rd tertile vs. 1st tertile	1.55 (0.53–4.51)		3.6 (1.22–10.64)*	2.98 (0.82‐10.82)	2.11 (0.80–5.59)	
Diet (discretionary pattern)						
2nd tertile vs. 1st tertile	3.11 (1.04–9.3)*	4.76 (1.17–19.26)*	1.07 (0.37–3.06)		0.73 (0.29–1.89)	
3rd tertile vs. 1st tertile	1.36 (0.46–4.03)	1.73 (0.41–7.38)	0.48 (0.17–1.39)		0.56 (0.21–1.50)	
Diet (industrialised pattern)						
2nd tertile vs. 1st tertile	0.75 (0.26–2.16)		2.86 (0.95–8.63)		2.88 (1.02–8.09)*	1.78 (0.58–5.50)
3rd tertile vs. 1st tertile	1.02 (0.38–2.73)		1.85 (0.59–5.82)		2.07 (0.72–5.93)	1.70 (0.54–5.30)
*Parent's characteristics*						
Parental perception on child's nutritional status (matched and overestimated vs. underestimated)	0.10 (0.03–0.30)**	0.06 (0.02‐0.21)**	0.21 (0.08–0.55)**	0.24 (0.08–0.74)*	0.45 (0.19–1.07)	

*Note*: OR, odd ratio (reference group is healthy/underweight children), aOR, adjusted odd ratio (adjusted for energy intake and physical activity and for each other).

* *p* < 0.05

** *p* < 0.001.

## 4 DISCUSSION

The overall prevalence of overweight and obesity among children aged 9–11 years old in Ho Chi Minh City was high (60% overweight and obese, 32% obese). A number of factors were associated with either overweight, obesity or overweight and obesity (combined) and elevated waist‐to‐height ratio at the child, familial and societal level. At the child and family level, being a boy, using screens for at least 2 h/day, having a father with obesity, being in a household in the lowest household income quintile, and having a parent who underestimates their child's weight status were found to be factors that were independently associated with increased odds of obesity, overweight or elevated waist‐to‐height ratio. Three major dietary patterns were derived. Traditional patterns were associated with obesity whereas discretionary patterns were associated with overweight and industrialized patterns were associated with elevated waist‐to‐height ratio.

At the societal level, household income was negatively associated with obesity after adjustment. This finding contradicted previous studies in Vietnamese children and adolescents, where obesity was more common in families with higher incomes (Carrillo‐Larco et al., 2016; Hong et al., 2007). However our results were more aligned with the finding of Weaver et al. (2019), who found that obesity was inequally high between the highest and the lowest income groups. Parents who attained higher levels of education and households with higher income were found to have more self‐efficacy to influence their child's dietary and physical activity practices (Supporting Information: Table S4), and potentially had more resources to provide their child with a healthier diet and access to after‐school physical activity programmes (To et al., 2018). In Vietnam, economic development and changes to the free trade agreement resulted in a decrease in the cost of food per one kilocalorie of dietary energy (Global Panel on Agriculture and Food Systems for Nutrition, 2016) as well as a remarkable reduction in price of sugary foods (Harris et al., 2020). As a result, energy dense, nutrient‐poor foods have become more accessible to low‐income families, with the rising prevalence of obesity being a consequence (Harris et al., 2020; Templin et al., 2019). The rapid increase in the prevalence of overweight and obesity in school‐aged children (5–19 years old) was previously identified in the National Nutrition Survey of Vietnam (National Institute of Nutrition, 2021); however, the disparity regarding SES in urban areas has not previously been examined. So, given the existence of inequitable distribution of childhood obesity in our sample, monitoring SES indicators in future nutritional surveillance surveys in Vietnam, particularly in urban areas would be beneficial. According to our findings, it is likely that Ho Chi Minh City is moving into stage 3 of the nutrition transition where prevalence of obesity in low‐income families is beginning exceed that in high‐income families (Jaacks et al., 2019). More rural areas are likely to still be in earlier stages of the nutrition transition where the prevalence of overweight and obesity was lower in rural districts compared to urban districts (Mai et al., 2020b) and undernutrition was still common in other parts of Vietnam (Harris et al., 2020). Consequently, it is not feasible to have a single national nutrition intervention programme but rather such programmes need to be tailored to the regional context.

At the family level, prevalence of obesity may be exacerbated in boys if the father is obese, given the strong association between father and child BMI in poorer households (Pham et al., 2020). In our study, a father's BMI ≥ 25 was strongly associated with obesity in the child, and obesity was more prevalent in boys. A likely relevant factor here is that there are strong cultural norms in Vietnam where boys are encouraged to be big and strong, which might potentially contribute to the disparity in prevalence of obesity between sexes (Sano et al., 2008). In addition, fathers are often missing from intervention programmes to control childhood overweight and obesity. For example, in a systematic review about the involvement of fathers in childhood obesity treatment and prevention, fathers only represented 6% of parents who participated (Morgan et al., 2017). Given the associations observed in our study, due consideration needs to be given to whole‐of‐family interventions that include fathers as a potential role models for their sons.

Another parent‐related factor that was associated with obesity in our study was parents' underestimation of their child's weight status, which was associated with an increased risk for childhood overweight and obesity. The prevalence of underestimation of child's weight status in our study were higher than the global percentage (55%) (Alshahrani et al., 2021) but was similar to other Asian parents (Park, 2017). A high proportion (>75%) of weight status underestimation by parents was found among children with overweight or obesity. This underestimation is likely to be a result of the rapid nutrition transition. It was once the norm to see a large proportion of undernourished children. Therefore, a chubby child was viewed as healthy or well‐nourished child (Do et al., 2016; Pan et al., 2021). We now know that these overweight children are at risk of cardiovascular disease (Mai et al., 2020a). So, there should be emphasis placed on early screening for overweight in Vietnamese children so that suitable interventions can commence early. Given the strong tracking of overweight and obesity from childhood to adulthood, early intervention has the potential to reduce to risk of obesity in adulthood (Pérez‐Escamilla & Kac, 2013; WHO, 2017).

In our study, parents who perceived their child as obese had a significantly lower score of self‐efficacy to influence their child's dietary practices and physical activity compared with parents who perceived their child's weight as normal (Supporting Information: Table S5). Given the association between parental perception of child's weight status and their self‐efficacy to influence on child's dietary practices and physical activities, these findings indicate that interventions could also focus on supporting parents in their understanding of their child's weight status, the relevance to their health, and on measures that would support parents in their self‐efficacy.

In Vietnam, the extended family is still common (41% of families in our sample) and grandparents often take care of children when their parents go to work. The perception of grandparents on child's weight status should be considered, given the fact that evidence from other studies has shown that a whole‐family‐based approach was successful in improving children's BMI and related behaviours by fostering parenting skills to support child's behaviours (Chai et al., 2019). Therefore, the involvement of all caregivers including father, mother, grandparents should be considered in the development of intervention programmes to manage children's weight and adjust social norms related to the child's weight status.

Finally, at the child level, dietary patterns were also associated with weight status. The unadjusted analyses showed that the traditional dietary pattern was associated with obesity, the discretionary pattern was associated with overweight and the industrialized pattern was associated with elevated waist‐to‐height ratio. However, attenuation of these associations occurred with multivariable adjustment and repeated analyses in a larger sample of Vietnamese children is needed to evaluate these associations.

In other studies, the sugar‐sweetened beverage and snack pattern characterised by fresh/favoured juice, flavoured milk drink, carbonated drinks, tea drinks, plant‐protein drinks, puffed foods, fried foods and Westerned fast foods (Min et al., 2021), or modern dietary pattern characterised by high intake of wheat, processed meat and fast foods (Zhen, 2018), or diets containing high amounts of obesegenic foods (such as fatty cheeses, sugary drinks, processed foods, fast food, etc.) were consistently associated with childhood overweight or obesity. Our findings also showed that the discretionary pattern, characterised by high intakes of sweetened beverages (two times/day) and snacks (including sweet, salty snacks) 1.5 times/day was positvely associated with overweight. The industrialized pattern, characterised by high intake of fast foods (five times/week) and processed meat pattern (five times/week) were positively associated elevated waist‐to‐height ratio (Supporting Information: Table S6). Elevated waist‐height ratio is an indicator of high central adiposity which is associated with increased risk of metabolic complications (Mai et al., 2020a). Early detection of children who are overweight with a central distribution of excess body fat would likely be beneficial in preventing metabolic complications of overweight and obesity. Improving children's dietary practices and enhancing parenting skills to shift children's dietary practices and physical activities toward healthier behaviours should be considered in intervention programmes that involve parents in obesity prevention and treatment.

With Vietnam experiencing the nutrition transition, sweetened beverages and discretionary foods have become more accessible due to high availability and low cost of these foods in convenience stores (Wertheim‐Heck & Raneri, 2019), in school canteens (Do et al., 2012) and supermarkets (Baker & Friel, 2016; Global Panel on Agriculture and Food Systems for Nutrition, 2016; Harris et al., 2022). Food marketing is also popular in Vietnam (Pham & Worsley, 2016). Although consumers are increasing their awareness about healthier diets, and companies have attempted to modify products toward healthier options, unhealthy, ultra‐processed foods continue to be readily available. Vietnam does not have regulations in place for controlling the sale and marketing of ultra‐processed foods (UNICEF Vietnam Country Office, 2021). Due to globalisation, Vietnam recently has signed numerous free trade agreements; however, optimising nutrition was not considered as part of these agreements except for concerns related to food safety. In addition, the consultation processes for this agreement were not transparent and did not have the opportunity for nutrition specialists to give comments or feedback (Harris et al., 2022). So, to mitigate the increased consumption of unhealthy diets, improved policies and leadership in public health should not only focus on downstream approaches for behaviour change among children and their families, but also upstream approaches with the need for increasing emphasis on development of public policies to improve the healthiness and sustainability of food systems and the food environment in Vietnam. Perhaps this can be achieved by higher taxes on certain ultraprocessed foods and sweetened beverages, regulation of marketing of unhealthy foods (high fat, sugar, salt), and the development of front‐of‐package labelling (UNICEF Producer, 2021). In addition, the contribution to nutrition and health should be considered in negotiation processes for international agreements and trade in particular around food. The commercial determinants of health should also be considered in developing nutrition policy, particularly as they relate to children's health and settings.

### 4.1 Strengths and limitations

This study has several strengths. First, the sample of participants is representative of children in Ho Chi Minh City with regard to area and ethnicity. The distribution was similar to Ho Chi Minh City's population, with 76% of participants living in urban districts and 96% of participants were Kinh ethnicity (Ho Chi Minh City Statistical Office, 2019). Second, all anthropometric indicators were measured twice by trained research assistants, which increased the validity and accuracy of measurements. Thirdly, this study used multi‐pass 24‐h recalls (two weekdays and one weekend) with trained assistants to capture children's usual diet and thus generated robust data of children's dietary intake. Finally, the dietary pattern analysis by PCA facilitated a food‐basedwhole‐of‐diet approach to dietary assessment, which takes into account the complex interactions between nutrients and bioactive compounds in the combination of foods usually consumed. Health communications based on such dietary patterns characterised by familiar foods are more easy to understand and more likely to be adopted than nutrient‐focused messages. Regarding the limitations, the sample size for this study, while statistically powered, was relatively small (*n* = 221) and the subsample for dietary data smaller again (*n* = 124). Thus, when examining the associations between dietary patterns and overweight and obesity, the wide CI of OR estimates indicate that precision was relatively low and type 2 errors may have occured. Examination of these associations with a larger sample size would be beneficial to verify the observed relationships or lack thereof. In addition, the self‐reported questionnaire about contributing factors to childhood overweight and obesity may have been influenced by a certain level of reporting bias, with parents potentially over‐or underreporting certain factors based on their perception of what the researchers were expecting. However, when considering the personal nature of some of these questions, self‐reported questionnaires such as those used in this study, are likely to be the preferred method for collecting such data, rather than interview administered. Finally, children's cognitive capacity may influence the accuracy of the reported diet (Smith et al., 2011). Children at 9 years old have been documented as able to report on their own diet (Baranowski et al., 1986); however, before the age of 12, the capacity to report portion size is potentially still limited (Livingstone et al., 2004). Thus, although this study had used several techniques to support children in reporting their diet such as photos of foods, common utensils and involved removing the under and overreporting records, bias in reporting diets may have still occurred.

## 5 CONCLUSION

Childhood overweight and obesity is highly prevalent among children 9–11 years old in urban Vietnam, particularly in boys, those with screen time > 2 h/day, father's obesity, parental underestimation of child's weight status, and in those living in the lowest income families. Dietary patterns appeared to be associated with either overweight, obesity or elevated waist‐to‐height ratio though not after confounder adjustment. Efforts targeting childhood obesity in urban Vietnam should consider childhood inequity and unhealthy food environment as key issues that need to be addressed. Parents should be supported in understanding and addressing their child's weight status which could include the involvement of fathers and grandparents to help improve dietary practices and physical activities of Vietnamese children.

## AUTHOR CONTRIBUTIONS

Thi My Thien Mai conducted the research. Thi My Thien Mai, Quoc Cuong Tran, Smita Nambiar, Danielle Gallegos, and Jolieke C. Van der Pols designed the research study. Thi My Thien Mai analysed the data. Thi My Thien Mai, Quoc Cuong Tran, Smita Nambiar, Danielle Gallegos, and Jolieke C. Van der Pols wrote the paper.

## CONFLICT OF INTEREST STATEMENT

The authors declare no conflict of interest.

## Supporting information

Supporting information.

## Data Availability

The data that support the findings of this study are available from the corresponding author upon reasonable request.
