# Supplementary material for: Dietary patterns and child, parental, and societal factors associated with being overweight and obesity in Vietnamese children living in Ho Chi Minh city
Source: Matern Child Nutr. 2023 Apr 3;20(Suppl 2):e13514. doi: 10.1111/mcn.13514 (PMC10984611; doi:10.1111/mcn.13514)
Supplement: Supplementary file 1 — Supporting information. [file MCN-20-e13514-s001.docx]

**Dietary patterns and child, parental, and societal factors associated with overweight and obesity in Vietnamese children living in Ho Chi Minh City**

Supplementary Table S1 Characteristics of under and over-reporters in the sub-sample with dietary data from three 24-hour recalls†

| Characteristic | Total Participants  (n = 163) | Under and Over  Reporters (n = 19) | Participants  (n = 144) |
| --- | --- | --- | --- |
| Sex, male | 81 (49.7) | 10 (52.6) | 71 (49.3) |
| Age (year) | 10.6 ± 0.5 | 10.8 ± 0.6 | 10.6 ± 0.5 |
| Weight (kg) | 41.5 (34.1–49.3) | 41.5 (33.7–52.7) | 41.5 (34.1–48.5) |
| Height (cm) | 143.2 ± 7.1 | 143.3 ± 7.2 | 142.9 ± 6.9 |
| BMI z-score (SD) | 1.25 (0.2–2.2) | 1.3 (0.3–2.2) | 1.3 (0.2–1.3) |
| *Nutritional status* |  |  |  |
| Thinness | 5 (3.1) | 1 (5.3) | 4 (2.8) |
| Normal | 62 (38.0) | 8 (42.1) | 54 (37.5) |
| Overweight | 46 (28.2) | 3 (15.8) | 43 (29.9) |
| Obesity | 50 (30.7) | 7 (36.8) | 43 (29.9) |
| Energy intake (kcal/day) | 1932 (1625–2232) | 2581 (1185–3285) | 1916 (1645–2178) |

† No significant difference between under and over reporters and participants

Supplementary Table S2 Food groups description

| Food groups | Description |
| --- | --- |
| Grains | Cereals, bread, root and starchy |
| Vegetables | Vegetables, legumes, and mushroom |
| Fruits | Fruits and fresh fruit juices without adding sugar |
| Meat and alternatives | Meat, seafoods, eggs, and beans, |
| Consuming dairy products | Plain milk, yogurt, and cheeses |
| Sweetened beverages | Added sugar beverages including added sugar milk |
| Snacks | Cakes, sweets, salty snacks |
| Fast foods | Pizza, hamburgers, fried dumplings/ potato |
| Instant noodles | Instant noodles |
| Processed meat | Sausage, bacon, ham, paste |

Supplementary Table S3 Dietary patterns derived from principal component analysis from frequency of food group intakes from three 24-hour recalls (N=124)

| Food groups | Traditional | Discretionary | Industrialized |
| --- | --- | --- | --- |
| Eating grains | 0.560 |  |  |
| Eating vegetables | 0.474 |  |  |
| Eating fruits |  |  |  |
| Eating meat and alternatives | 0.467 |  |  |
| Consuming dairy products |  |  | - 0.341 |
| Drinking sweetened beverages |  | 0.669 |  |
| Eating snacks |  | 0.585 |  |
| Eating fast food |  |  | 0.568 |
| Eating instant noodles | -0.341 |  |  |
| Eating processed meat |  |  | 0.609 |
| % variance explained | 23 | 17 | 13 |
| Eigenvalue | 2.301 | 1.645 | 1.329 |

Loadings based on the assigned scores from the food groups generated from three 24-hour recalls; factor loading with values greater or equal to 0.3 were considered and included in the pattern. Blanks cells represent food groups with loading values <0.3

Supplementary Table S4 Parental’s self-efficacy score by parental education level*

| Parental self-efficacy score to influence children's eating practices and activities | | Total score (max = 140) | |  | Eating score (max = 80) | |  | PA score (max = 60) | |
| --- | --- | --- | --- | --- | --- | --- | --- | --- | --- |
|  |  | Mean | SD |  | Mean | SD |  | Mean | SD |
| **Father education** | |  |  |  |  |  |  |  |  |
|  | Seconary school or lower (n=89) | 83.8 | 34.7 |  | 50.7 | 20.8 |  | 33.1 | 16.7 |
|  | High school or higher (n=81) | 100.6 | 25.5 |  | 60 | 15.4 |  | 40.6 | 13.2 |
| **Mother education** | |  |  |  |  |  |  |  |  |
|  | Seconary school or lower(n=111) | 84.1 | 34.1 |  | 50.7 | 20.9 |  | 33.4 | 16.3 |
|  | High school or higher(n=57) | 101.4 | 24.3 |  | 60 | 14.6 |  | 41.4 | 12.4 |

*p<0.05 Student t-test to examine the difference between education level among father an mother

Supplementary Table S5 Parental’s self-efficacy and parental perception on child’s weight status

| Parental's perception of child's weight status | | Total participants (n = 161) | |  | Healthy weight and underweight (n= 107) | |  | Overweight (n=32) | |  | Obese (n=17) | |  | Overweight and obesity (n=49) | | |
| --- | --- | --- | --- | --- | --- | --- | --- | --- | --- | --- | --- | --- | --- | --- | --- | --- |
|  |  | **Mean** | **SD** |  | **Mean** | **SD** |  | **Mean** | **SD** |  | **Mean** | **SD** |  | **Mean** | **SD** |  |
|  | Total score (max = 140) | 93.4 | 30.1 |  | 96.1 | 29.6 |  | 94.1 | 27.0 |  | 78.2 | 38.7* |  | 88.6 | 32.0 |  |
|  | Eating score (max = 80) | 56.2 | 18.1 |  | 57.5 | 18.1 |  | 57.1 | 16.9 |  | 47.9 | 20.8* |  | 53.9 | 18.6 |  |
|  | PA score (max = 60) | 37.3 | 14.9 |  | 38.6 | 13.7 |  | 37.0 | 16.0 |  | 30.3 | 19.8* |  | 34.7 | 17.5 |  |

*p<0.05, Student T-test examine the difference of parental self-efficacy score between who perceived their child as overweight, or obesity, or overweight and obesity and healthy weight and underweight

Supplementary Table S6 Energy intake and frequency intake of each food groups for each dietary pattern by terciles (N=124)

| Food groups | Traditional pattern | | |  | Discretionary pattern | | |  | Industrialised pattern | | |  |
| --- | --- | --- | --- | --- | --- | --- | --- | --- | --- | --- | --- | --- |
|  | Q1 | Q2 | Q3 | P-value | Q1 | Q2 | Q3 | P-value | Q1 | Q2 | Q3 | P-value |
| Energy intake | 1813(346) | 1929(358) | 2055(374) | 0.001 | 1762(334) | 1888(315) | 2148(357) | <0.001 | 1836(308) | 1943(386) | 2018(397) | 0.026 |
| Eating grains | 2.5(0.5) | 3.0(0.3) | 3.9(0.7) | <0.001 | 3.4(0.8) | 3.1(0.8) | 2.8(0.7) | 0.002 | 2.9(0.7) | 3.1(0.6) | 3.4(0.9) | 0.008 |
| Eating vegetables | 1.4(0.5) | 2.0(0.4) | 2.5(0.5) | <0.001 | 2.0(0.7) | 1.9(0.8) | 2.0(0.6) | 0.846 | 1.8(0.7) | 2.0(0.6) | 2.0(0.7) | 0.201 |
| Eating fruits | 0.7(0.5) | 1.0(0.4) | 1.3(0.6) | <0.001 | 0.9(0.5) | 0.8(0.5) | 1.2(0.6) | 0.026 | 1.1(0.6) | 1.0(0.5) | 0.9(0.6) | 0.348 |
| Eating meat and alternatives | 2.5(0.4) | 3.1(0.5) | 3.7(0.5) | <0.001 | 3.1(0.7) | 3.1(0.7) | 3.1(0.7) | 0.771 | 3.0(0.7) | 3.2(0.6) | 3.1(0.7) | 0.328 |
| Drinking dairy | 0.1(0.2) | 0.2(0.3) | 0.2(0.3) | 0.037 | 0.2(0.3) | 0.2(0.2) | 0.2(0.3) | 0.956 | 0.3(0.4) | 0.1(0.2) | 0.1(0.2) | 0.005 |
| Drinking sweetened beverages | 2.3(1.0) | 1.9(1.0) | 2.5(1.3) | 0.896 | 1.4(0.6) | 2.1(0.6) | 3.2(1.0) | <0.001 | 2.1(1.0) | 2.0(0.9) | 2.5(1.3) | 0.258 |
| Eating snacks | 1.5(0.9) | 1.5(0.8) | 1.7(1.0) | 0.802 | 0.9(0.5) | 1.5(0.7) | 2.4(0.9) | <0.001 | 1.8(1.0) | 1.4(0.8) | 1.4(1.0) | 0.025 |
| Eating fast food | 0.4(0.4) | 0.4(0.4) | 0.3(0.3) | 0.297 | 0.4(0.4) | 0.4(0.4) | 0.4(0.3) | 0.540 | 0.2(0.2) | 0.3(0.3) | 0.7(0.4) | <0.001 |
| Eating instant noodles | 0.5(0.4) | 0.2(0.3) | 0.1(0.3) | <0.001 | 0.1(0.2) | 0.2(0.3) | 0.4(0.4) | 0.001 | 0.2(0.3) | 0.3(0.3) | 0.4(0.4) | 0.081 |
| Eating processed meat | 0.3(0.4) | 0.4(0.3) | 0.6(0.4) | 0.002 | 0.5(0.3) | 0.5(0.3) | 0.4(0.4) | 0.110 | 0.2(0.2) | 0.4(0.3) | 0.7(0.3) | <0.001 |

Data was presened as mean(sd)
